# Multidimensional profile of functional cognitive disorders: the experience of the memory clinic in Abidjan, Côte d’Ivoire, sub-Saharan Africa

**DOI:** 10.3389/fneur.2026.1836371

**Published:** 2026-07-08

**Authors:** Kouamé Léonard Kouassi, Assi Viviane Yao, N’guessan Yves Constant Broh, Nawa Samuel Yéo, Ange Stéphane Abbé, Affoué Marie Roxane Beuseize, Ahya Nancy Tanya Essoin-De Souza, Ismaïla Diakité, Mariam Doumbia-Ouattara

**Affiliations:** 1 Faculty of Medical Sciences, Felix Houphouet-Boigny University, Abidjan, Côte d'Ivoire; 2 Department of Neurology, University Hospital of Yopougon, Abidjan, Côte d'Ivoire

**Keywords:** Abidjan, functional cognitive disorder, internal inconsistency, memory consultation, memory impairment, mild neurocognitive disorder

## Abstract

**Background:**

Functional cognitive disorders (FCD) are increasingly recognized but underdiagnosed. In sub-Saharan Africa, the topic is rarely discussed and the data is unknown. The goal of this study was to examine the epidemiological, clinical, and neuropsychological aspects of FCD in order to contribute to a better understanding of this etiological entity.

**Methods:**

This was a retrospective, multicenter, descriptive and analytical study. From 2018 to 2023, we included patients at least 18 years old, with no abnormalities in neurological examination and brain magnetic resonance imaging, presenting an internal inconsistency between reported symptoms and signs identified in neuropsychological evaluation. Cognitive deficits were assessed using the Montreal Cognitive Assessment (MoCA) test, which identifies three levels of impairment: mild (score 18–25), moderate (score 10–17), and severe (score < 10).

**Results:**

During the study period, 25 cases of FCD were identified, representing 10.2% of the total. The most represented age group was 45–54 years old (52%) with an average age of 45.8 years. More than half of our patients (56%) had a university education level, and most of them worked in the public sector (48%) and the private sector (36%). Mental health disorders (52%) were the most common comorbidities. Memory disorders were the most common reason for consultation (72%). The mean MoCA test score was 23.2 ± 5.9. The cognitive abilities most affected were memory (44%), attention (28.3%), and visuospatial/executive function (28%). There were no sociodemographic or clinical factors associated with the patients’ cognitive performance.

**Conclusion:**

This study, which is the first of its kind in Côte d’Ivoire and one of the few in sub-Saharan Africa on this topic, could help expand and improve knowledge about FCD in sub-Saharan Africa, where data is extremely scarce, particularly in Côte d’Ivoire.

## 1 Introduction

As the global population ages, neurocognitive disorders will affect more and more people. Their prevalence is higher in high-income regions, such as Western Europe, than in Asia and Africa (1). Neurocognitive Disorders of Neurodegenerative Origin are the most common, with Alzheimer’s disease being the most prevalent (2, 3). On the other hand, non-neurodegenerative causes, including functional cognitive disorders (FCD), rare and account for approximately 12% of the causes (4).

Although still underdiagnosed among neurocognitive disorders, FCD are increasingly recognized as a distinct entity (5). As a result, memory clinics are seeing an increasing number of patients with this condition, and approximately 25% of patients visiting these clinics for suspected dementia may in fact have a FCD (6). Its prevalence varies between 10 and 76% depending on the study (7). FCD are characterized by significant subjective cognitive complaints without corresponding objective evidence of neurological pathology (5). The mechanisms involved are thought to involve psychological factors, attentional dysfunctions, and impaired metacognitive control, rather than structural brain damage (5).

Diagnosing FCD can be complex, and misdiagnosis of early-stage neurodegeneration is possible. However, a set of operational diagnostic criteria clinically useful for FCD was proposed by Ball et al. (5). These criteria include one or more symptoms of cognitive impairment; evidence of functional impact, reflected by avoidance of cognitively demanding tasks in social situations, despite preserved objective performance; evidence of internal inconsistency in performance; symptoms that are not better explained by another medical, neurological, or psychiatric disorder; and symptoms that cause significant distress, impairment in functioning, or require clinical attention. Despite this, the diagnosis of FCD must always be refined (5).

In Africa, data on FCD are scarce, and research on dementia or neurocognitive disorders tends to focus on Alzheimer’s disease, vascular dementia, and HIV/AIDS-associated neurocognitive disorders (8). However, a misdiagnosis of FCD could lead to unnecessary investigations, inappropriate treatments, and increased anxiety in the patient. Conversely, a correct diagnosis reassures the patient and allows targeted interventions, such as psychoeducation, cognitive-behavioral therapy, and metacognitive training, which have been shown to be effective in improving patient prognosis (5).

In Côte d’Ivoire, neurocognitive disorders are still not well known in general, but particularly FCD. Furthermore, there are few specialists in cognition, not to mention the difficulties in accessing a memory consultation. However, FCD require special attention for a rigorous diagnosis due to their diagnostic complexity, which can be confused with other pathologies such as mild cognitive impairment, pseudodementia, and dementia. Our goal was to study the epidemiological, clinical, and neuropsychological characteristics of this etiological entity of neurocognitive disorders in our work context, in order to better understand it, avoid diagnostic delays, and quickly relieve patients.

## 2 Patients and methods

### 2.1 Study design and framework

In Côte d’Ivoire, memory clinics are a relatively recent phenomenon. As a result, neither healthcare professionals nor the general public are very familiar with them. These consultations are not routine; they are conducted on request by neurologists specializing in neuropsychology. This study was therefore conducted at two private referral centers in Abidjan where “memory clinics” are held. This is a retrospective, descriptive, and analytical study that examined patient records from 2018 to 2023.

### 2.2 Study population and sampling

The study focused on patient records received in “Memory Consultation” in the aforementioned structures.

Patients included in this study were at least 18 years old, had no abnormalities on neurological examination and magnetic resonance imaging, had an internal inconsistency between reported symptoms and signs identified in neuropsychological evaluation, and had no neurocognitive disorder explained by an organic or psychiatric cause.

The sampling was exhaustive by recruiting all cases of FCD observed during the study period.

### 2.3 Data collection

Data were collected using a structured questionnaire that covered the sociodemographic, clinical, and neuropsychological aspects of the patients.

The sociodemographic aspects included age, sex, handedness, occupation, marital status, and educational level.

The clinical aspects included the reason for the consultation, the patient’s medical history, and the history of the symptoms.

The neuropsychological variables included data from the Montreal Cognitive Assessment (MoCA) and the Diagnostic and Statistical Manual of Mental Disorders, 5th Edition (DSM-5).

The MoCA data were characterized by the mean MoCA score, the MoCA sub-scores and the average level of impairment (lost proportion) and the average preserved capacity (preserved proportion) of each cognitive function.

The MoCA sub-scores are defined as follows: Visuospatial/executive function score (out of 5 points), Naming score (out of 3 points), Attention score (out of 6 points), Language score (out of 3 points), Abstraction score (out of 2 points), Memory score (out of 5 points) and Orientation score (out of 6 points).

The average level of impairment of a cognitive function (lost proportion) is the average number of points lost (as a percentage) out of the total points for that function. The average number of points remaining (as a percentage) represents the average preserved capacity (preserved proportion) for that function.

The DSM-5 data included mild neurocognitive disorder and major neurocognitive disorder.

### 2.4 Evaluation of neurocognitive functions

The neuropsychological aspects were obtained using the “Montreal Cognitive Assessment (MoCA), French version 8.3” (9) and the “Diagnostic and Statistical Manual of Mental Disorders. 5th Edition (DSM-5)” (10) tests.

The MoCA was designed to assess mild cognitive dysfunction. It evaluates the following functions: attention, concentration, executive functions, memory, language, visuoconstructive abilities, abstract thinking, calculation, and orientation.

The MoCA can be administered by anyone who understands and follows the instructions. However, the result can only be interpreted by a healthcare professional with expertise in cognition. The execution time is approximately 10 min. The maximum number of points is 30; a score of 26 or more is considered normal (9).

A score below 26 indicates neurocognitive impairment with three levels:

- A score between 18 and 25 indicates a mild impairment;
- A score between 10 and 17 indicates a moderate impairment;
- A score strictly below 10 indicates severe impairment.

The DSM-5 defines two types of neurocognitive disorders: major neurocognitive disorder, characterized by the presence of neurocognitive disorder associated with impaired autonomy; and mild neurocognitive disorder marked by the presence of neurocognitive disorder with preserved autonomy (10).

### 2.5 Statistical analysis

Data were recorded in CSPRO version 7.3 software and then exported to SPSS 26 software for statistical analysis. The quantitative variables were expressed by their means if the distribution of the variables followed a normal law. The graphs were edited using Microsoft Office Excel 2013 software and the tables using Microsoft Office Word 2013.

To investigate associations between the independent variables (sociodemographic and clinical) and the dependent variable (MoCA scores), we performed a univariate analysis using a nonparametric test, Fisher’s exact test, due to the small sample size. A *p*-value of <0.05 was considered statistically significant.

We will denote “ns” for *p*-values that are not statistically significant (*p* ≥ 0.05).

## 3 Results

### 3.1 Descriptive aspect

#### 3.1.1 Epidemiological characteristics

**Figure 1:**
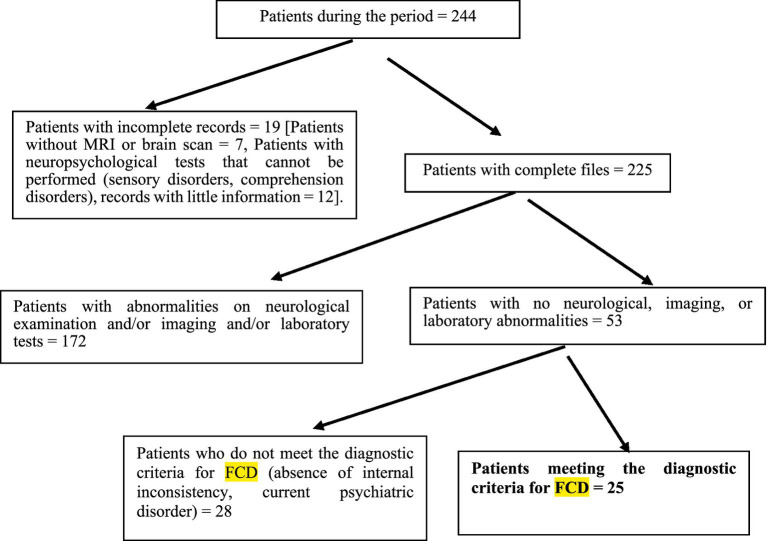
FCD patient selection process.

Between September 2018 and December 2023, we enrolled 244 patients, of whom 25 (10.2%) met the inclusion criteria (Figure 1). The study sample consisted of 13 men and 12 women (sex ratio: 1.1). The average age of the patients was 45.8 ± 11 years with extremes of 26 and 69 years. The levels of education were mainly represented by university (56%) and secondary (44%) levels. The profession was dominated by the public (48%) and private (36%) sectors. The sociodemographic characteristics of the patients are described in Table 1.

**Table 1:** Sociodemographic characteristics of patients.

Variables	Effective	Percentage (%)
Sexes		
Men	13	52
Women	12	48
Age groups		
<50	15	60
≥50	10	40
Education levels		
Not schooled	0	0
Primary	0	0
Secondary	11	44
University	14	56
Occupations		
Public sector	12	48
Private sector	9	36
Other occupations^a^	4	16

^a^Liberal sector = 1, Students = 1, Retirees = 1, Unemployed = 1.

#### 3.1.2 Clinical characteristics

The average time from the onset of neurocognitive impairment to the first day of the memory consultation was 29.9 months, with extremes of 1 and 120 months. The majority of patients (72%) were referred to a memory clinic by a physician, and memory disorders were the most common reason (72%). Mental health issues (52%) were the most common history, with depression (36%) and anxiety or stress (16%) followed by high blood pressure (24%). The patients’ clinical data are represented in Table 2.

**Table 2:** Clinical characteristics of patients.

Variables	Effective	Percentage (%)
Consultation requests		
Addressed by a doctor	18	72
Came on his/her own	12	24
Accompanied by a loved one	1	4
Complaints		
Memory problems	18	72
Other complaints^a^	3	12
Cognitive status	4	16
Medical history		
Depression	9	36
Anxiety or stress	4	16
High blood pressure	6	24
Others^b^	9	36

^a^Language disorders (4%), incoherent statements (4%), behavioral disorders (4%). ^b^Diabetes (4%), Tobacco (4%), herniated disc (8%), diabetes (4%), tobacco (4%), sickle cell disease (4%), gout (4%), functional colopathy (4%).

#### 3.1.3 Neuropsychological characteristics

The mean MoCA score was 23.2 ± 5.9 with extremes of 26 and 69. In 36% of cases, the MoCA score was normal and mild impairment (18 ≤ score ≤ 25) was predominant (48%). The majority of patients had mild neurocognitive disorder (92%), Table 3.

**Table 3:** Distribution of patients by MoCA score and type of neurocognitive disorder.

Variables	Effective (n)	Percentage (%)
MoCA score level		
Normal ≥26	9	36
Mild (18 ≤ score ≤ 25)	12	48
Moderate (10 ≤ score ≤ 17)	2	8
Severe (score < 10)	2	8
Neurocognitive disorders (DSM-5)	Effective (n)	Percentage (%)
Mild	23	92
Major	2	8

**Figure 2:**
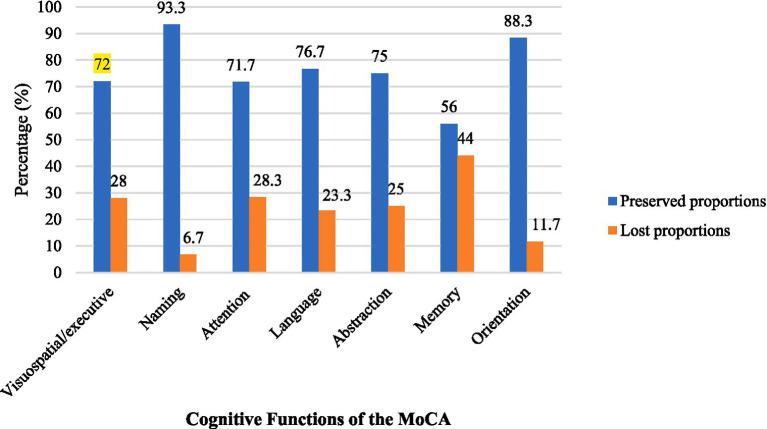
Average level of impairment (lost proportion) and average preserved capacity (preserved proportion) of cognitive functions.

Figure 2 illustrates the average level of impairment (lost proportion) and the average preserved capacity (preserved proportion) of cognitive functions. Memory had the highest level of impairment (44%) of all functions. It was followed by attention and visuospatial/executive function in 28.3 and 28% of cases, respectively. In contrast, naming and orientation had the highest levels of preserved capacity in 93.3 and 88.3% of cases, respectively.

### 3.2 Analytical aspects

Statistical analysis of the data to identify an association between the independent variables (sociodemographic factors and medical history) and the dependent variable (MoCA score) revealed no significant association between these variables, with *p* > 0.05. The results are shown in Table 4.

**Table 4:** Bivariate analysis between sociodemographic factors, medical history, and MoCA category (normal vs. abnormal).

Variables	MoCA score				*p*-value
	Normal		Abnormal		
	n	(%)	n	(%)	
Age (year)					ns
<50	6	24	9	36	
≥50	3	12	7	28	
Sexes					ns
Men	5	20	8	32	
Women	4	16	8	32	
Education levels					ns
Not Schooled	0	0	0	0	
Primary	0	0	0	0	
Secondary	2	8	9	36	
University	7	28	7	28	
Occupations					ns
Public sector	4	16	8	32	
Private sector	4	16	5	20	
Other occupations	1	4	3	12	
Mental disorders					ns
Yes	7	28	6	24	
No	2	8	10	40	
High blood pressure					ns
Yes	2	8	4	16	
No	7	28	12	48	

## 4 Discussion

The objective of this study was to investigate the epidemiological, clinical, and neuropsychological characteristics of FCD in memory clinics in Abidjan to contribute to a better understanding of this etiological entity of neurocognitive disorders.

The prevalence of FCD was 10.2%. There was no gender predominance. The patients were generally young, with an average age of 45 years. They all had either a university or secondary education. These patients worked primarily in the public and private sectors. Patients consulted a memory center late (average delay: 29.9 months).

Clinically, patients were most often referred by a physician, and the most common complaints were memory problems. The medical history was dominated by psychiatric disorders, followed by High blood pressure.

Neuropsychologically, patient performance was good (84%), with 36% of patients having a normal MoCA score and 48% having mild impairment. In 92% of cases, patients met the criteria for mild neurocognitive disorder (DSM-5). The cognitive abilities most affected were memory, attention, and executive functions. No sociodemographic or clinical associations were observed with MoCA test performance.

### 4.1 Epidemiological characteristics

The prevalence observed in our study falls within the range described in the literature, with a prevalence of 10–76% (7). Pennington et al. in the United Kingdom in 2015 reported 23 cases of FCD in 196 patients examined, a prevalence of 11.7% (11). In Brazil in 2017, Gondim et al. noted a prevalence of FCD in 13.6% of cases (12). Our literature review does not mention any data from sub-Saharan Africa, reflecting a lack of knowledge on this subject. Furthermore, since the diagnostic criteria are not clearly established, the prevalence found in our study may be underestimated.

The average age of our patients was 45.8 years, and only one patient was over 65 (69 years). Barhambe et al., in the United Kingdom, in a study on FCD in memory consultation, found an average age of 51.7 years (13). In a recent literature review, Cabreira et al. showed that patients with FCD were younger than those with neurodegenerative neurocognitive disorders (7).

Our study found no difference in terms of gender, although 52% of the FCD cases involved men. This result differs from those in the literature, which note a sex ratio in favor of women, particularly in the series of Stone et al., where FCD was diagnosed twice as often in women as in men (14). This discrepancy may be related to the size of our sample, which appears to be small.

The level of education of our patients was high, consistent with that observed by Cabreira et al., who reported that patients with FCD were more educated, tended to have more education, and were more often university graduates (7). Education has a recognized protective effect on cognitive decline, particularly in people who had more than 10 years of education; and higher education level is a protective factor against dementia and a more suggestive indicator of FCD (7, 15, 16).

The professional sectors most represented in our study were the public sector (48%) and the private sector (36%). Indeed, in these sectors, employees are salaried and are therefore required to consult a doctor in the event of a health problem in order to justify their absence from work. Bhome et al. also reported a predominance of salaried patients (1).

### 4.2 Clinical characteristics

Our patients presented late at memory clinics, with an average delay of two and a half years. This delay reflects a lack of understanding of cognitive disorders in general, and DCF in particular, among healthcare staff and the general public. This delay may be one of the consequences of a lack of awareness. Furthermore, there is a severe shortage of memory specialists, and memory centers are very rare. Added to this are the sociocultural considerations specific to our populations in the African context regarding neurocognitive disorders in general (17). This delay could also be linked to these patients’ preserved social and professional autonomy despite their cognitive complaints. Furthermore, DCF progress in a stable, non-progressive manner and show no signs of converting into dementia (5). They are also characterized by periods of fluctuation. Consequently, memory loss and concentration difficulties may be considered part of the normal aging process or attributed to fatigue.

Memory disorders were the main reason for consultation in our study (72%). Among the established diagnostic criteria for FCD, memory disorders are listed as one of the most common symptoms (18, 19). In the 2013 series by Rock et al., patients with FCD had a more significant impairment in the domains of memory and executive functions (20).

Mental health disorders, particularly depression and anxiety, were the most common comorbidities found in our study (52%). Depression and FCD can coexist, and Bhome et al. reported depression associated with FCD in 49% of cases (21). A higher prevalence of primary psychiatric disorders, mainly anxiety and depressive disorders (36–55%), was observed in patients with FCD (7).

### 4.3 Neuropsychological characteristics

Our study reported a mean MoCA score of 23.2, which defines the mild stage of MoCA interpretation. Pennington et al. in 2019 in the United Kingdom and McWhirter et al. in the United Kingdom reported similar results, 23.9 and 22 (22, 23), respectively, which corroborates our data. This score reflects the good performance of our patients in the neuropsychological evaluation, contrasting with their complaints that were debilitating. Indeed, the majority of these were addressed by a doctor testifying to the severity of their complaints. Internal inconsistency in the FCD often manifests as moderate to severe cognitive disorders in the patient, but with milder deficits in cognitive tests (24). Furthermore, according to the DSM-5, the majority of our patients (92%) had mild neurocognitive disorder with preserved autonomy. Literature data reveal that FCD is characterized by higher MMSE scores in cognitive exams than patients with mild cognitive impairment, dementia, or other cognitive disorders (7). Furthermore, nine of our patients (9/25, 36%) scored within the normal range on the MoCA test (score ≥ 26/30), similar to the score reported by Pennington et al. in the United Kingdom (7/23, 39%) (11).

The cognitive abilities most affected in our study were memory, attention, and visuospatial/executive function. Regarding the FCD, the cognitive domains that most frequently show poor performance on neuropsychological tests are those related to attention, concentration, and executive functions (21). Although there is no consistent data available on the cognitive profile of patients with FCD and on the differences with other neurodegenerative diseases, some studies demonstrate the importance of evaluating cognitive domains such as memory, attention, information processing speed, and executive functions using tests that are sensitive enough to detect potential deficits (25, 26). The MoCA test, commonly used to detect mild neurocognitive disorders, revealed significant memory impairment in our study. Wakefield et al. showed that the performance of patients with FCD did not differ from that of healthy controls, except in tests of attention and executive function, in which the control group performed better than the FCD group (27).

### 4.4 Factors associated with FCD

In our study, no sociodemographic, psychiatric, or vascular factors were significantly associated with cognitive performance. Although younger age and higher educational attainment were more common among patients with FCD than among those with cognitive disorders of degenerative origin, none of these factors is specific to FCD (6). Furthermore, the small size of our sample could explain the lack of an association. This suggests that further studies with larger samples are needed to confirm these findings.

Regarding psychiatric factors, the literature indicates that FCD is generally associated with psychiatric disorders, such as depression and anxiety, which may even constitute risk factors or comorbidities (7). However, depression is not a statistically significant predictor of the decline in objective cognitive deficits, particularly deficits in verbal learning and memory (28). These psychiatric factors can instead be recognized as risk factors for major neurocognitive disorders (29). As for vascular risk factors, they are rarely associated with FCD (7). However, Gondim et al. noted in their study an association between FCD and hypertension (12).

### 4.5 Strengths and weaknesses of the study

Due to the small size of our sample, the strength of the analysis may be weak. It is also important to note that the size of our sample is not the first, as other studies have found smaller samples. Pennigton et al. reported samples of 23 and 21 patients with FCD in 2015 and 2019, respectively (11, 23). However, this study has the merit of contributing to improving knowledge about FCD in sub-Saharan Africa, where data is extremely scarce, particularly in Côte d’Ivoire.

## Data Availability

The original contributions presented in the study are included in the article/supplementary material, further inquiries can be directed to the corresponding author.
